# Helping global health topics go viral online

**DOI:** 10.7189/jogh.10-010101

**Published:** 2020-06

**Authors:** Iain H Campbell, Igor Rudan

**Affiliations:** Centre for Global Health Research, Usher Institute for Population Health Sciences and Informatics, The University of Edinburgh, Scotland, UK

## Abstract

To maintain support for further investments into health research and prevent large groups of people from questioning modern science, researchers will increasingly need to master their communication of scientific progress in the 21st century to broad general population. The new generations, who inform and educate themselves online, prefer to make their own choices in what they view. This makes them vulnerable to many types of online misinformation, which is placed there mainly to attract their clicks. This evolving context could strongly undermine a consensus in the population over very important public health issues and gains. Therefore, we believe that it deserves to be recognised as a serious problem of our time that needs to be addressed. In addition to possible inaccuracies of the health information found online, the other component of the problem is how to make global public health topics and issues attractive for viewing online and engaging with. They need to compete with popular music, celebrity gossip, sports, movies and other forms of entertainment. In this issue, we bring a novel series aiming to explore effective strategies to promoting global health issues online and through other mass media, and reaching wide audiences.

Global health is a rapidly evolving field of biomedical science, but it also has a social, political and economic dimension that tends to capture people's imagination. This makes global health research amenable to community engagement and popularization among general public. However, it is difficult to know which strategies would work best to attract a large number of viewers to video materials that convey accurate global health information. This is particularly important in recent years, where it became apparent that any reliable information online could soon get its seductive, but inaccurate counterpart in an effort to enhance someone's personal “click bait” rate [1]. Humans seem to be quite receptive to alternative views, especially when they are surprising, shocking or spectacular, or when they seem to be contrary to long-held popular beliefs or common knowledge [1,2]. Such news always tends to be more interesting to broad population than the stories that merely consolidate or confirm the existing knowledge [1,2]. But this also poses a potential danger when the creators of alternative and fake news “attack” an important public health issue and cause confusion among the population, thus risking reversal in many hard-achieved gains in population health [3]. Anti-vaccine movements and are probably the most striking example of this risk [4].

Increasingly, the scientists involved in global health, but also all other areas of science, are beginning to realise that moving forward and generating more knowledge for humanity is not our only task; in fact, it may no longer be our main task, either. Communicating the knowledge that has been generated to broad spectrum of people in a convincing and accurate way may be just as important, if not more [4,5]. Otherwise, we are risking a new phenomenon: that those who generate new knowledge become completely isolated from the large majority of the human population, the later no longer understanding the results of the science properly, and showing a propensity for moving backwards as a consequence of reading fake news and doubtful information found on the internet [4,5].

We need to seriously consider approaches that have been effective in spreading various misinformation and fake news on the internet, countering the scientific knowledge achieved through many years of incredibly hard work and rigorous experimental science, and underpinned by the most modern technology available to researchers [4,5]. Only through mastering our communication of scientific progress in the 21st century to broad general population, we can expect support for further research and prevention of masses of people questioning modern science and going backwards in time.

However, most scientists are not at all trained in communicating the results of their research to massive, large audiences of lay people in general population. Moreover, research has shown that scientists are not clear about how does public engagement fit into their job [6,7]. There are often no benchmarks or rewards for this work, it takes a lot of time while distracting them from the work they must do (such as raising grants and writing papers), and they often worry how would such engagement affect their reputation among their colleagues [6,7].

In this issue, we bring a novel series aiming to explore effective strategies to promoting global health issues online and through other mass media, and reaching wide audiences. This is particularly important in view that the new generations have grown up mainly using the internet as their source of information, communication and entertainment. They no longer watch traditional television or listen to the radio programmes, where the information was historically more likely to be scrutinized, particularly when it comes to public health messages. The new generations prefer to make their own choices in what they view. Relying on the internet makes them vulnerable to misinformation of various sorts, placed there mainly to attract their clicks. This could strongly undermine a consensus in the population over very important public health issues and gains, and it deserves to be recognised as a serious problem of our time that may still evolve and needs to be properly addressed in some way.

In addition to the problem of the accuracy of the information on global health found online, the other side of this problem is how to make global health topics and issues attractive for viewing and engaging with, in competition with popular music, celebrity gossip, sports, movies and other forms of entertainment. In an iconic attempt to make the topic of global maternal and child mortality “sexy”, Save the Children famously organised an audition for New York's best-looking male and female models and asked them to read out into the camera the facts about maternal and child mortality in their most seductive voice, making an excellent point how difficult it is to make those topics attractive to general audiences [8].

However, there were some wonderful success stories in conveying global health issues to wide audiences online. The hero for so many of us, the late Dr Hans Rosling, was arguably the most famous statistician in recent decades due to his remarkable efforts in visualising extremely complex and multi-dimensional data and presenting them in a way that was engaging and educational for the widest masses [9]. He proved to everyone that even something so unthinkable can be done, but it took his genius and a lot of remarkable effort. His short film “200 Countries, 200 Years, 4 Minutes – The Joy of Stats”, aired by BBC 4, accumulated more than 8 millions views on YouTube to date [10], while his famous TED talk “Debunking third-world myths with the best stats you've ever seen” was also seen by millions of people [11].

Several other efforts also reached notable viewership online in promoting global health issues. Global Health Media Project develops educational videos for frontline health workers everywhere in the world. In collaboration with the International Federation of Red Cross and Red Crescent Societies, UNICEF, and Yoni Goodman, they developed a cartoon “The Story of Ebola”, which served as an educational material on this epidemic and it was seen by more than 6 million viewers [12]. Their earlier film, “The Story of Cholera”, and another one on the breastfeeding in the first hours after birth, were seen by more than 3 million people to date [13]. Another notable success through the use of animated stories was achieved by the Global Health Workforce Alliance (GHWA), in partnership with bliinktv in the UK, which developed a beautiful animated film called “Imagine...” on the shortage of health workers in many of the poorest world countries and settings [14]. It has been seen by more than 150 000 people. Other notable efforts are a YouTube channel by the Editor-in-Chief of the journal “Globalization and Health”, Dr Greg Martin, who produced nearly a 100 videos on various global health topics and attracted more than 19 000 subscribers to his channel [15]. There is now also a Global Health TV channel on YouTube with more than 140 videos posted, and also Duke University's Global Health institute's channel, with more than 440 videos posted.

Still, these successful efforts are very rare outliers. There are less than 100 videos on YouTube today focused using “global health” as the search term that have been seen by 10000 viewers or more. But this does not mean that the general public is not interested in global health topics. They will be interested, but not in the language that scientists would use, but rather in mainstream media labels given to some “vertical” issues and problems. For example, the terms “superbugs”, “antibiotic apocalypse”, “zika”, etc., would not be found under “global health” search term, but there are videos on these topics that are hugely popular and attracted millions of viewers. This sends an important message that, if scientists are to engage with massive viewership online, they need to acquire an understanding for the language that is being used in the mainstream media to “label” global health issues, rather than stick to their scientific vocabulary.

Many of the highly popular videos became popular probably because they exhibited some of the key features defined by Jonah Berger and Katherine L. Milkman is their study “What Makes Online Content Viral?” [16]. In this issue, we will present our own attempt to develop a viral educational content on global health – a Massive Open Online Course at the University of Edinburgh, called “Survival: The Story of Global Health” [17]. In a series of papers we'll document our strategies and experiments that we conducted in order to explore how to make an effective global health community engagement material that could go “viral” online, similarly to some more popular topics on YouTube and other platforms.
